# Herpes Simplex Virus 1 (HSV-1) Infected Cell Protein 0 (ICP0) Targets of Ubiquitination during Productive Infection of Primary Adult Sensory Neurons

**DOI:** 10.3390/ijms24032931

**Published:** 2023-02-02

**Authors:** Telvin L. Harrell, David J. Davido, Andrea S. Bertke

**Affiliations:** 1 Biomedical and Veterinary Science, Virginia-Maryland College of Veterinary Medicine, Virginia Polytechnic Institute and State University, Blacksburg, VA 24060, USA; 2 Molecular Biosciences, University of Kansas, Lawrence, KS 66045, USA; 3 Population Health Sciences, Virginia-Maryland College of Veterinary Medicine, Virginia Polytechnic Institute and State University, Blacksburg, VA 24060, USA; 4 Center for Emerging Zoonotic and Arthropod-Borne Pathogens, Virginia Polytechnic Institute and State University, Blacksburg, VA 24060, USA

**Keywords:** HSV, human herpes virus, alphaherpesvirus, ICP0, mass spectrometry, primary neurons, HMG I/Y, High Mobility Group Protein I/Y, TDP43, TAR DNA Binding Protein 43

## Abstract

Herpes simplex virus 1 (HSV-1) enters sensory neurons with the potential for productive or latent infection. For either outcome, HSV-1 must curtail the intrinsic immune response, regulate viral gene expression, and remove host proteins that could restrict viral processes. Infected cell protein 0 (ICP0), a virus-encoded E3 ubiquitin ligase, supports these processes by mediating the transfer of ubiquitin to target proteins to change their location, alter their function, or induce their degradation. To identify ubiquitination targets of ICP0 during productive infection in sensory neurons, we immunoprecipitated ubiquitinated proteins from primary adult sensory neurons infected with HSV-1 KOS (wild-type), HSV-1 *n*212 (expressing truncated, defective ICP0), and uninfected controls using anti-ubiquitin antibody FK2 (recognizing K29, K48, K63 and monoubiquitinated proteins), followed by LC-MS/MS and comparative analyses. We identified 40 unique proteins ubiquitinated by ICP0 and 17 ubiquitinated by both ICP0 and host mechanisms, of which High Mobility Group Protein I/Y (HMG I/Y) and TAR DNA Binding Protein 43 (TDP43) were selected for further analysis. We show that ICP0 ubiquitinates HMG I/Y and TDP43, altering protein expression at specific time points during productive HSV-1 infection, demonstrating that ICP0 manipulates the sensory neuronal environment in a time-dependent manner to regulate infection outcome in neurons.

## 1. Introduction

Herpes simplex virus 1 (HSV-1) is a double-stranded DNA virus that infects an estimated 66.6% of the global population [1]. HSV-1 infections can cause recurring orofacial and genital lesions [2], resulting in pain, itching, and discomfort for the host [3], as well as stress and anxiety in the affected individual. In some instances, however, HSV-1 can cause additional complications such as severe skin manifestations, herpetic keratitis [4], and life-threatening encephalitis [5]. Furthermore, HSV-1 has been implicated in the development of neurodegenerative diseases, including Alzheimer’s disease [6,7]. The recurrence pattern, frequency, and chances of more severe complications vary significantly between individuals. Treatment options are limited to a guanosine analog, acyclovir and its derivatives, which can be instrumental in reducing the severity or frequency of HSV-1 recurrences, but an HSV-1 infection cannot be cured and individuals remain infected for life.

The pathogenesis of HSV-1 is complex, with an infection cycle that begins in epithelial cells and progresses to peripheral sensory and autonomic neurons [8]. In epithelial cells, HSV-1 follows a temporal cascade of gene expression consisting of immediate-early, early, and late genes. Immediate-early (IE) genes modulate the intracellular host environment to be more conducive to viral infection and initiate transcription of viral early and late genes. Early (E) genes mediate the synthesis of new viral DNA strands, with further manipulation of the host environment. Late (L) genes are predominantly structural, forming the viral capsid and coordinating viral DNA packaging into progeny virions. Completion of this temporal cascade results in the production of mature virions and the demise of the host cell. In neurons, however, the temporal cascade can be altered depending on the neuronal phenotype. Peripheral sensory ganglia contain a heterogenous population of neurons that respond to different stimuli and neurotrophic factors; they also differentially regulate HSV-1 infection [9,10,11]. In some neurons, HSV-1 will progress through a productive infection while in others, the virus will establish latency. Latency is a period of viral quiescence in which the viral genome will persist indefinitely and may reactivate in response to various stress-inducing insults. The mechanisms that regulate the decision to undergo productive infection or establish latency after entry into neurons are unclear.

HSV-1 expresses five IE proteins that contribute to the early stages of productive infection. Each IE protein antagonizes different aspects of the host cell, with molecular redundancy and some functional overlap. Infected cell protein 0 (ICP0) and infected cell protein 22 (ICP22) modify the intracellular host cell environment by ubiquitinating host proteins [12] and altering the physiology of the nucleus [13], respectively. Infected cell protein 47 (ICP47) inhibits the transporter associated with antigen processing (TAP) protein complex, preventing MHC class I presentation of HSV-1 antigens to immune cells [14,15], contributing to viral evasion. Infected cell proteins 4 (ICP4) and 27 (ICP27) interfere with host cell gene expression to curtail host antiviral responses while increasing viral gene expression [16,17,18]. Collectively the IE proteins modulate host cell functions at multiple levels to establish the foundation necessary for HSV-1 infection. Whether these proteins function to promote productive infection or the establishment of latency upon viral entry into sensory neurons is not clear.

ICP0 and its functions have been extensively studied in non-neuronal cells. Through experiments conducted in HeLa cells, fibroblasts, and Vero cells, ICP0 has been shown to downregulate proinflammatory mechanisms [19] and toll-like receptor signaling [20], interfere with cell cycle regulation [21], increase viral transcription [22], and decrease viral genome silencing [23,24]. ICP0 also has the ability to activate promoters of IE, E, and L genes [22] without binding DNA or RNA directly. However, it is unknown if these functions identified in non-neuronal cells translate to mature neurons, where HSV-1 establishes latency. ICP0 is classified as an E3 ubiquitin ligase, containing a ubiquitin ligase domain associated with a RING (Really Interesting New Gene) finger domain [25,26]. As such, ICP0 can direct the last step of the ubiquitin cascade, catalyzing the addition of ubiquitin moieties to target substrates. Ubiquitination facilitates downstream effects such as degradation by the proteasome or redirecting a target protein for another function. ICP0 has been shown to interact with an array of proteins, such as the deubiquitinating enzyme ubiquitin-specific peptidase 7 (USP7) [27] and cellular E2 ubiquitin-conjugating enzymes UbcH5a and UbcH6a [28] to regulate its ubiquitination functions [29]. The proteins known to be ubiquitinated in the presence of ICP0 in non-neuronal cells include essential cellular proteins such as p53 [30], Sp100, USP7 [27], and Schlafen 5 [24] but in neurons, where HSV-1 can undergo either productive or latent infection, the ubiquitination targets of ICP0 remain unknown.

To identify sensory neuron-specific ubiquitin targets of HSV-1 ICP0 during productive infection, we utilized an anti-ubiquitin antibody (FK2) to immunoprecipitate ubiquitinated proteins from primary adult sensory neurons infected with either wild-type HSV-1 KOS or HSV-1 *n*212, which expresses a truncated, defective form of ICP0. Identification of the ubiquitinated proteins by mass spectrometry and comparison between the infected and uninfected neurons identified neuronal proteins specifically targeted for ubiquitination by ICP0, providing insight into neuron-specific molecular mechanisms regulated by ICP0 during HSV-1 infection. We selected two of these proteins, High-Mobility Group Protein I/Y (HMG I/Y) and TAR-DNA Binding Protein 43 (TDP43), for further study.

## 2. Results

### 2.1. ICP0 Protein Profile Is Biphasic in Primary Adult Sensory Neurons

**Figure 1:**
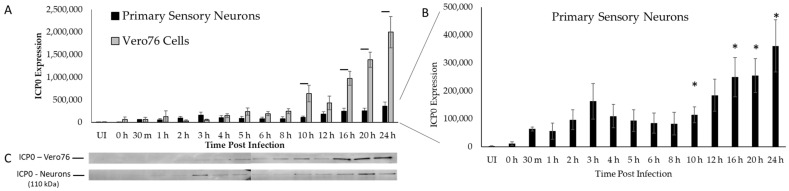
Protein expression profile of ICP0 in Vero 76 cells and primary adult sensory dorsal root ganglion (DRG) neurons (**A**), based on densitometry analysis, normalized to fluorescent detection of total protein per lane using 2,2,2-trichloroethanol (TCE) for visualization and densitometry (*n* = 4 independent immunoblots). To show the pattern of expression of ICP0 in sensory neurons over time, the sensory neuron expression data in A is also shown on a modified scale ((**B**), note Y-axis). Representative Western blots are shown (**C**). UI = uninfected neurons, 0 h = immediately upon inoculation. Error bars = SEM. Bars (**A**) and asterisks (**B**) = statistically significant difference between Vero76 cells and primary sensory neurons (*p* < 0.05).

ICP0 expression patterns and protein levels have been determined previously through sparsely collected time points using a variety of non-neuronal cell types and modified in vitro assays [21,31,32,33]. Although essential information has been obtained using these methods, mechanisms in non-neuronal cell types likely differ from those in neurons, where HSV-1 manipulates the environment to either proceed through productive infection or establish latency and yet remain poised to reactivate. To determine the ideal time to perform mass spectrometry to identify potential ICP0-mediated ubiquitin targets, we needed to produce a clear and concise protein expression profile for ICP0 during productive infection in primary adult sensory neurons. Since previous assessments were performed in non-neuronal cells, we assessed ICP0 expression in non-neuronal cells in parallel as a comparison. Vero76 cells and cultured primary adult dorsal root ganglion (DRG) neurons were inoculated with wildtype HSV-1 KOS strain, and independent samples of each were collected incrementally over 24 h, beginning at time 0 (T0), immediately upon inoculation (Figure 1). In Vero76 cells, ICP0 was first detected above 0 h levels as early as 1 h post inoculation (hpi) and steadily increased through 24 hpi with minor fluctuations (Figure 1A). ICP0 protein expression was significantly greater in Vero76 cells compared to expression in neurons at 10 hpi, and 16–24 hpi (*p* < 0.05. In contrast, ICP0 was detected above 0 h levels 30 min post inoculation in adult sensory neurons and increased to a distinct minor peak 3 hpi (Figure 1B). ICP0 protein levels subsequently decreased until 8 hpi, after which ICP0 increased through 24 hpi, the last time point we analyzed (Figure 1B). Representative Western blots are shown (Figure 1C).

This biphasic protein profile of ICP0 appears to be unique to neurons, suggesting dynamic mechanisms and the possibility of multiple functions at different times during productive HSV-1 infection. Early events, during the first “wave” of ICP0 expression, likely contribute to the decision between productive infection vs. establishment of latency in sensory neurons. As our goal was to identify the greatest possible number of proteins that are ubiquitinated by HSV-1 ICP0 early during productive infection, we selected the 8 h time point to identify and compare ubiquitinated proteins in the neurons. Although protein expression declined from 3–8 hpi, ICP0 was still present and presumably still functional. By treating the neurons with the proteasome inhibitor MG132, we were able to prevent degradation of ubiquitinated proteins until the time of collection. This approach allowed us to identify any proteins that were ubiquitinated within the first 8 h of HSV-1 infection in primary sensory neurons.

### 2.2. Mass Spectrometry Analysis of Proteins Ubiquitinated by ICP0

**Figure 2:**
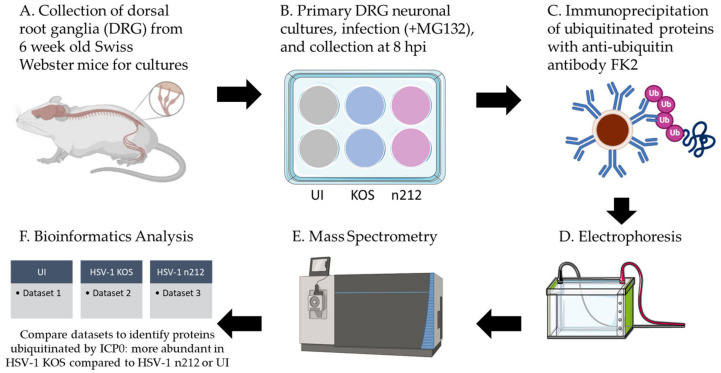
Schematic representation of the sample preparation for LC-MS/MS. (**A**) Primary adult dorsal root ganglion (DRG) neurons were resected from 6 wk old Swiss Webster mice and cultured at a minimum of 40,000 neurons per well. Two wells per condition were analyzed during each of two experiments. (**B**) DRG neurons were treated with MG132 and infected with HSV-1 KOS or HSV-1 *n*212 (30 MOI), or left uninfected (UI) for 8 h. (**C**) Equal amounts of total DRG protein were mixed with FK2 antibodies covalently bound to magnetic Dynabeads to immunoprecipitate ubiquitinated proteins from each sample. Proteins were eluted from beads. (**D**) Protein eluate was separated on a 10% SDS-PAGE gel, excised into 10 equal size bands, and digested in-gel with trypsin. (**E**) Peptides were analyzed by nano LC-MS/MS. (**F**) Data were analyzed and compared.

ICP0 engages in complex protein–protein interactions to modify the cellular environment during productive infection [26]. Some of ICP0’s interactions catalyze the transfer of ubiquitin to target proteins for proteasomal degradation [34], while others stabilize ICP0 [35]. Several protein interactors, such as Sp100 nuclear antigen [36], PML (promyelocytic leukemia protein) [36], and USP7 (ubiquitin-specific-processing protease 7) [37] have been identified in U2OS cells, human embryonic lung fibroblasts, and other non-neuronal cell types, but proteins targeted by ICP0 for ubiquitination in neurons remain unknown. To identify proteins that ICP0 selectively ubiquitinates in primary adult sensory neurons, we utilized a comparative mass spectrometry approach, illustrated in Figure 2. We infected primary adult dorsal root ganglion (DRG) sensory neurons with HSV-1 KOS, which is a wildtype virus that expresses fully functional ICP0, or HSV-1 *n*212, which expresses a non-functional ICP0 fragment due to a nonsense linker inserted at codon 212 built in a KOS background [38]. Proteins ubiquitinated by ICP0 would be enriched in KOS-infected neurons but would not be ubiquitinated, or would be ubiquitinated at a lower rate, in uninfected samples or HSV-1 *n*212-infected samples because ICP0 is absent or non-functional, respectively.

Primary cultured DRG neurons from 6-week-old mice were infected with KOS or *n*212 in the presence of MG132, a cell-permeable proteasome inhibitor, to prevent degradation of any proteins ubiquitinated before collection (Figure 2). Uninfected neurons were maintained in parallel and also treated with MG132. HSV-1 infection was allowed to progress for 8 h before total protein from each condition was collected in a non-denaturing buffer with proteasome inhibitor MG132, deubiquitinase inhibitor PR619, and protease/phosphatase inhibitors to protect the conjugated ubiquitin moieties on target proteins. Equal amounts of protein from each condition were incubated overnight with magnetic beads conjugated to FK2 antibodies that recognize K29, K48, and K63 poly-ubiquitination chains and mono-ubiquitinated proteins [39]. Immunoprecipitated samples were analyzed by nano liquid chromatography tandem mass spectrometry (LC-MS/MS) to generate datasets of putative proteins ubiquitinated in each condition.

**Figure 3:**
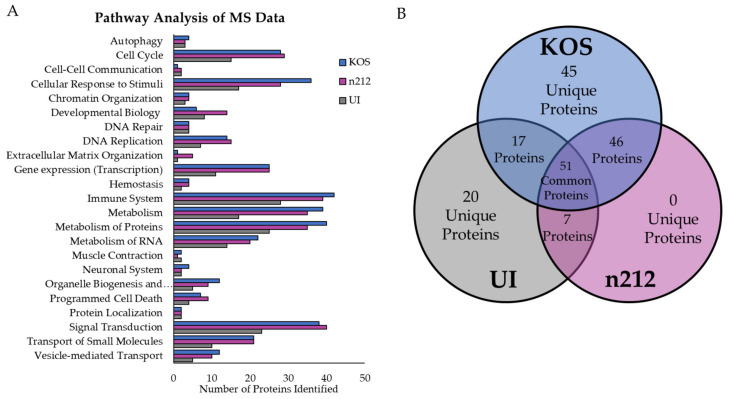
Protein classification of ubiquitinated proteins identified by LC-MS/MS. (**A**) Proteins identified from neurons infected with HSV-1 KOS (wild type) or *n*212 (expressing truncated ICP0), or from uninfected (UI) neurons were classified based on known function through pathway analysis using Reactome. (**B**) Statistical analysis (ANOVA and post hoc Dunnett’s) identified 169 unique proteins: 45 unique to KOS-infected neurons, 20 unique to uninfected (UI) neurons, and proteins common to two conditions or all three.

Proteins were identified by at least 2 unique peptides and screened based on MASCOT score (≥50) and Exp-q score ≤ 0.05 for acceptable confidence in the protein identification. Based on these screening criteria, we classified the ubiquitinated proteins in each condition (KOS, *n*212, UI) for function and signaling pathway affiliation using Reactome (Figure 3A). Identified proteins mapped to biological pathways such as the cell cycle, cellular response to stimuli, immune system, cellular metabolism, and signal transduction, suggesting broad impacts on cellular processes during HSV-1 infection of neurons. To identify those proteins ubiquitinated by ICP0, we compared peptide spectrum matches (PSM) of each protein and focused on those proteins with at least a 1.25-fold increase in KOS-infected neurons when compared to HSV-1 *n*212-infected or uninfected neurons. In total, 169 unique host proteins and 17 viral proteins were identified. Of those, 30 host and 15 viral proteins were specifically ubiquitinated by ICP0 (identified only in KOS-infected neurons) (Figure 3B). We also identified 46 proteins in common between KOS- and *n*212-infected neurons, 26 of which were ubiquitinated at least 1.25-fold higher in KOS-infected compared to *n*212-infected neurons. Of the 17 common proteins identified in both uninfected (UI) and KOS-infected neurons, only one was more highly ubiquitinated by ICP0 (1.86-fold higher in KOS-infected neurons), but 13 were more highly ubiquitinated in uninfected neurons compared to KOS-infected neurons. Twenty (20) unique proteins were also identified only in uninfected (UI) neurons. These data suggest that the presence of the virus somehow inhibits ubiquitination of several host proteins, while simultaneously facilitating the ubiquitination of other host and viral proteins.

Both viral and host proteins were identified in our LC-MS/MS analysis of ubiquitinated proteins, providing insight into potential ICP0 ubiquitination targets as well as neuronal viral mechanics (Table 1 and Supplemental Table S1). ICP0 was identified in HSV-1 KOS-infected neurons, suggesting that ICP0 targets itself for ubiquitination early during productive infection in neurons. Other viral proteins were also detected only in KOS-infected neurons, including major viral transcription factor ICP4, tegument protein VP16, transcriptional regulator ICP22, and envelope glycoprotein B. These viral proteins were not identified in *n*212-infected neurons, suggesting that these proteins are selectively ubiquitinated by ICP0 during wild-type infection (Table 1. Viral Proteins). Thymidine kinase (TK) and the large subunit of ribonucleoside-diphosphate reductase were also ubiquitinated at significantly higher levels in KOS-infected compared to *n*212-infected neurons. As many of these viral proteins are important during productive infection, identification as ubiquitination targets in KOS-infected neurons suggests that ICP0 may be attempting to inhibit productive infection, at least in some sub-populations of the sensory neurons, early after infection.

The identified host proteins exhibited a broad range of functions, subcellular locations, and potential relevance to HSV-1 pathogenesis (Table 1. Host Proteins). Proteins such as cell cycle exit and neuronal differentiation protein 1 (CEND1), histone H1.2, 14-3-3 protein epsilon, and β-synuclein were enriched in KOS-infected neurons when compared to *n*212-infected and uninfected neurons, suggesting selective targeting by functional ICP0. Our focus, however, centered on transcription factors involved in gene expression regulation, gene repression, and neuron-specific functions that could be relevant to HSV-1 pathogenesis. Of the identified proteins, High Mobility Group Protein I/Y (HMG I/Y) and Trans-Activation Response (TAR) DNA-Binding Protein 43 (TDP43) fit these criteria. HMG I/Y is a member of the HMG superfamily consisting of three primary classifications: HMGA, HMGB, and HMGN [40,41]. The HMGA family, with which HMG I/Y is associated, utilizes AT-hook to alter the structure of DNA by binding inside the minor groove [42,43], making DNA more or less accessible to transcription factors and DNA binding proteins [44]. HMG I/Y has previously been shown to interact with TAATGARAT sequences within the HSV-1 genome, increasing viral gene expression [45], but its ubiquitination status in relation to HSV-1 has not been determined. TDP43 has not been linked to HSV-1, but was first identified for its role in binding to Human Immunodeficiency Virus (HIV) Trans-Activation Response (TAR) elements and repressing viral replication [46]. More recently, TDP43 has been shown to regulate spatiotemporal and tissue-specific gene expression, RNA polymerase pausing, and RNA splicing [47], impacting more than 30% of the cell transcriptome [48]. HMG I/Y and TDP43 are essential for neuronal gene expression and RNA metabolism, respectively. Given the previously reported role of these proteins in viral replication, we selected these proteins for further study to determine if their ubiquitination is mediated by ICP0, and their roles in HSV neuronal pathogenesis.

### 2.3. HMG I/Y and TDP43 Exhibit Increased Ubiquitination in the Presence of Functional ICP0

Ubiquitination of target proteins occurs through the addition of ubiquitin moieties on lysine residues on the target proteins [49]. To validate that ICP0, HMG I/Y, and TDP43 are ubiquitinated by ICP0 during HSV-1 productive infection, we inoculated primary DRG neuronal cultures with KOS or *n*212. After 8 h of infection, neurons were collected in a non-denaturing buffer with MG132 and PR619 to prevent proteasomal degradation and de-ubiquitination of proteins while in solution. Ubiquitinated proteins were subsequently immunoprecipitated using the FK2 antibody, used previously for mass spectrometry, followed by immunoblots using antibodies specific for HMG I/Y and TDP43. We also immunoblotted for ICP0 since ICP0 has been previously reported to self-ubiquitinate in cell-free assays and we detected ICP0 as a ubiquitinated protein in KOS-infected neurons by mass spectrometry, but not in neurons infected with *n*212 [50]. Total protein input (InP), supernatant from the immunoprecipitation (S), and the eluate from the FK2-conjugated beads (IP) were loaded onto SDS-PAGE gels and probed for each protein of interest using protein-specific antibodies.

**Figure 4:**
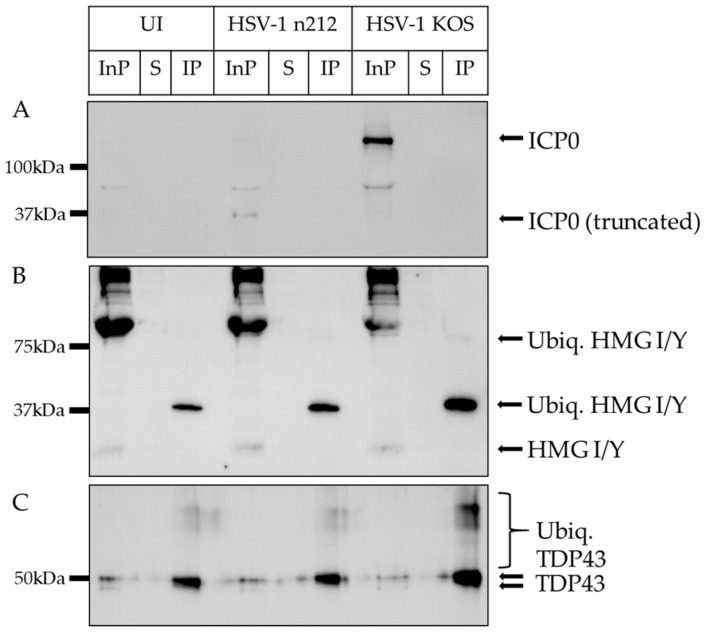
Ubiquitination of ICP0 (**A**), HMG I/Y (**B**), and TDP43 (**C**). Immunoprecipitation was conducted 8 hpi using Dynabeads conjugated to anti-FK2 antibodies that specifically targets K29, K48, K63, and mono-ubiquitinated proteins. Total protein input (InP), supernatant from immunoprecipitation (S) and immunoprecipitated eluate (IP) from uninfected (UI), HSV-1 *n*212-infected, and HSV-1 KOS-infected neurons were probed with antibodies targeting each protein of interest.

In our immunoprecipitation experiments, ICP0 was detected in the total protein input (InP) of KOS-infected neurons (~118 kDa), and the truncated ICP0 was detected in *n*212-infected neurons (~37 kDa) (Figure 4A). However, no ICP0 protein was detected in the supernatant, suggesting that the anti-ubiquitin FK2-conjugated beads had captured ICP0, but no detectable patterns of mono-ubiquitination or poly-ubiquitination were observed in the IP. HMG I/Y appeared at ~20 kDa in total protein (InP) samples and at ~37 kDa in IP samples (Figure 4B). HMG I/Y was not detected in the supernatant, demonstrating successful immunoprecipitation with FK2, and exhibited increased band density in HSV-1 KOS-infected samples. This shift in size is consistent with a change of approximately 17 kDa, supporting the addition of two ubiquitin moieties (8.6 kDa per ubiquitin) [51]. A faint band was also detected at ~80 kDa in KOS-infected neurons, suggesting a small portion of HMG I/Y was poly-ubiquitinated, as well (Figure 4B). TDP43 was detected as a doublet at 43 and 50 kDa in InP samples and a portion of the 50 kDa band was detectable in the supernatant (Figure 4C). These data indicate the likely presence of post-translationally modified forms of TDP43 in total protein (InP) samples, besides ubiquitination. In IP samples, TDP43 exhibited characteristic patterns of poly-ubiquitination with increased smear density above 50 kDa in KOS-infected samples. These results support that HMG I/Y and TDP43 are ubiquitinated by ICP0 during the first 8 h of productive HSV-1 infection in sensory neurons, but ubiquitinated ICP0 was not detected in this assay.

### 2.4. Productive HSV-1 Infection Alters HMG I/Y and TDP43 Proteins Profiles in Sensory Neurons

To determine if HMG I/Y and TDP43 are targeted for degradation during productive HSV-1 infection, mediated by ICP0, we analyzed the protein profile of each protein during productive infection with either KOS or *n*212 in the absence or presence of MG132 to inhibit the proteasome, preventing the degradation of ubiquitinated proteins. If HMG I/Y or TDP43 is degraded during productive infection, mediated by ICP0, we would observe a decrease in the respective protein only in samples infected with KOS without MG132. In comparison, HMG I/Y or TDP43 would be increased in KOS-infected samples treated with MG132 since the proteasome could not degrade the ubiquitinated proteins. These protein changes should not occur in *n*212-infected neurons because this truncated form of ICP0 is defective.

**Figure 5:**
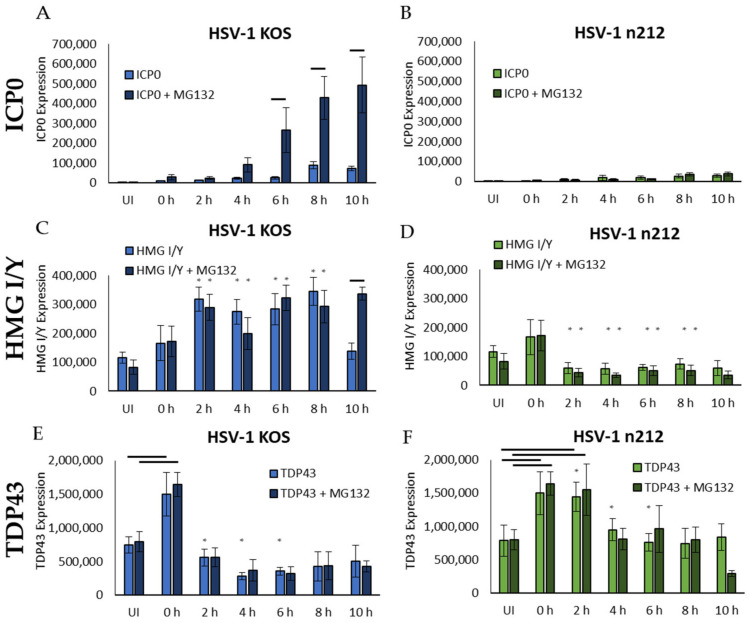

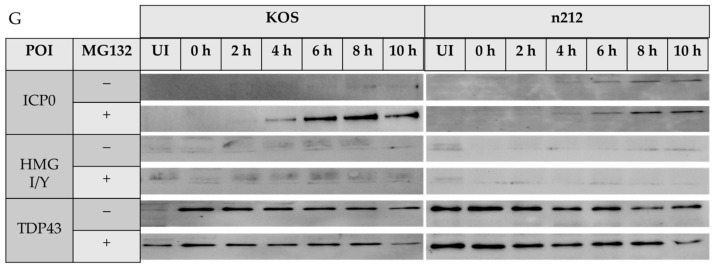
Protein expression of ICP0 (**A**,**B**), HMG I/Y (**C**,**D**), and TDP43 (**E**,**F**) during 10 h of HSV-1 KOS and HSV-1 *n*212 productive infection in primary adult DRG neuronal cultures, with or without MG132 to inhibit proteasomal degradation. Neurons were assessed every 2 h by Western blot (*n* = 3), analyzed by densitometry, and normalized to total protein visualized with TCE. Representative Western blots are shown (**G**). Error bars = SD. Horizontal bars indicate statistical significance between MG132 treated and untreated (**A**,**C**) or between 0 h and 2 h and UI (**E**,**F**) (*p* < 0.05). Asterisks indicate statistical significance between KOS- and *n*212-infected neurons (*p* < 0.05).

Primary adult DRG neuronal cultures were infected with KOS or *n*212, with or without MG132. Neurons were collected in 2 h increments for 10 h and immunoblotted for ICP0, HMG I/Y, or TDP43. Protein bands from three independent blots were quantified using densitometry, normalized to total protein visualized with stain-free, fluorescent detection of 2,2,2-trichloroethanol (TCE) included in the gel, and presented as bar graphs (Figure 5A–F) with representative immunoblot images (Figure 5G). ICP0 was detected at 0 hpi in KOS-infected neurons with and without MG132, indicating that ICP0 was present at low levels within the inoculating virus (Figure 5A), which is consistent with previous reports that ICP0 is present within the tegument of purified HSV-1 [52]. ICP0 protein expression levels in KOS-infected neurons without MG132 remained relatively low, but neurons treated with the proteasome inhibitor MG132 contained significantly greater quantities of ICP0 by 6 hpi (*p* < 0.05, Figure 5A). In contrast, truncated ICP0 expressed by *n*212 remained low and relatively consistent for neurons that were treated or untreated with MG132 (Figure 5B). These results demonstrate that full-length ICP0 is degraded by the proteasome during productive infection. Truncated ICP0 is not well-expressed, and is not degraded by the ubiquitin-proteasome pathway during productive infection in adult sensory neurons.

HMG I/Y was detected in uninfected neurons, increasing slightly but not significantly in response to inoculation (0 h compared to uninfected, Figure 5C,D), which was likely recognized as a transient stressor to the neurons. In KOS-infected neurons, HMG I/Y increased significantly compared to uninfected neurons by 2 hpi and remained high in both MG132 treated and untreated neurons (*p* < 0.05, Figure 5C). At 10 hpi, HMG I/Y protein level remained high in KOS-infected neurons treated with MG132 but was significantly decreased in untreated neurons, returning to uninfected levels (*p* = 0.55 compared to uninfected, Figure 5C), showing that HMG I/Y was degraded by the proteasome at this time point when ICP0 was present. In *n*212-infected neurons, HMG I/Y returned to uninfected levels by 2 hpi, suggesting that the impaired productive infection kinetics of *n*212 did not influence the overall level of HMG I/Y in adult sensory neurons and protein expression remained similar to uninfected neurons (*p* = 0.18–0.30, Figure 5D). Treatment with MG132 had no significant effects on HMG I/Y in *n*212-infected neurons, compared to untreated neurons, showing that HMG I/Y is not degraded by the proteasome when a non-functional ICP0 is expressed.

TDP43 protein was also detected in uninfected neurons and increased in response to virus inoculation at 0 h for both KOS and *n*212 infection (Figure 5E,F). In contrast to HMG I/Y, however, the initial increase in TDP43 levels was statistically significant compared to uninfected neurons (*p* < 0.05), and resolved within 2 h to levels similar to uninfected neurons in KOS-infected neurons, remaining stable and consistent for both MG132 treated and untreated neurons throughout the 10 h time period (Figure 5E). In *n*212-infected neurons, TDP43 remained high a little longer, for at least 2 h (*p* < 0.05). TDP43 then decreased to levels comparable to uninfected neurons by 4 hpi, remaining consistent through 8 hpi in neurons with and without MG132 (Figure 5F). At 10 hpi, in neurons treated with MG132, TDP43 decreased slightly but not significantly to a level below what was observed in uninfected neurons. This decrease was not observed in *n*212-infected neurons without MG132, in which the ubiquitin–proteasome is free to degrade ubiquitinated proteins. Al-though TDP43 is ubiquitinated by ICP0, it does not appear to be degraded in the proteasome, since MG132 had no significant effects on TDP43 protein expression. However, TDP43 protein expression was maintained at a significantly lower level during productive HSV-1 KOS infection between 2 and 6 h post infection (*p* < 0.05, indicated by asterisks) when compared to *n*212 infection, suggesting that HSV-1 infection results in reduced TDP43 expression early during productive infection when full-length ICP0 is expressed.

### 2.5. HMG I/Y Is Increasingly Ubiquitinated and Degraded by ICP0 between 8 and 10 hpi

**Figure 6:**
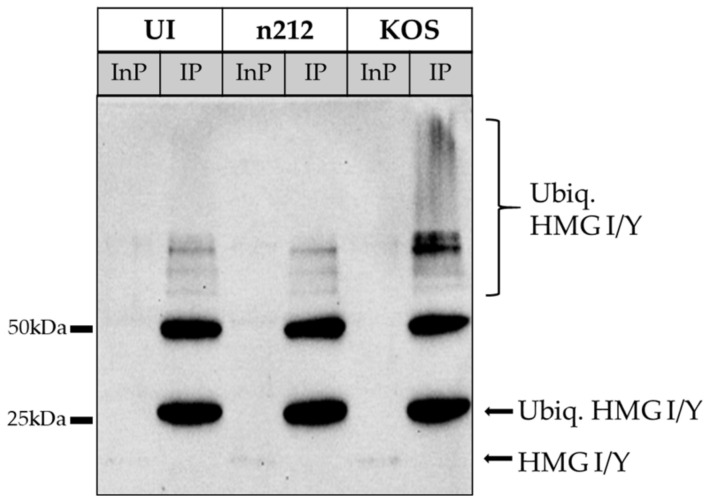
Ubiquitination of HMG I/Y. Immunoprecipitation was conducted 10 hpi using Dynabeads conjugated to anti-FK2 antibodies that specifically target K29, K48, K63, and mono-ubiquitinated proteins. Total protein input (InP) and immunoprecipitated eluate (IP) from uninfected (UI), HSV-1 *n*212-infected, and HSV-1 KOS-infected neurons were probed with antibodies targeting HMG I/Y.

HMG I/Y protein levels increased and remained high during the first 8 hpi in neurons infected with KOS, but decreased significantly at 10 hpi in KOS-infected neurons without MG132, which inhibits the proteasome (Figure 5C). The decrease suggested that in the presence of ICP0, HMG I/Y was degraded in the proteasome at 10 hpi. Our validation assay for ubiquitination was performed at 8 hpi and showed that only a small portion of HMG I/Y was ubiquitinated (faint band ~80 kDa in Figure 4B). To determine if ICP0 increasingly ubiquitinates HMG I/Y at 10 hpi beyond what we previously observed at 8 hpi, we infected primary adult sensory neurons with KOS or *n*212 in the presence of MG132, as described previously, and allowed the infection to progress for 10 hpi. KOS-infected, *n*212-infected, and uninfected neurons were collected in a non-denaturing buffer with MG132 plus inhibitors and immunoprecipitated using the FK2 antibody. Total protein input (Inp) and IP samples were immunoblotted to visualize patterns of ubiquitin for HMG I/Y. HMG I/Y exhibited bold patterns of ubiquitination in KOS-infected neurons at 10 hpi (Figure 6), correlating with the decrease in protein levels observed in KOS-infected neurons without MG132 at 10 hpi (Figure 5C). These data show that HMG I/Y is increasingly ubiquitinated by ICP0 between 8–10 hpi in the presence of wild-type ICP0, resulting in proteasomal degradation by 10 hpi.

## 3. Discussion

HSV-1 entry into a neuron can result in productive infection or the establishment of latency, depending on the type and physiological state of that neuron at the time of entry [9,53,54,55]. Previous studies have shown that host factors, such as chromatin mediators [56,57,58,59,60] and innate defense factors [24,61,62,63,64,65], contribute to the establishment of latency, but a clear mechanism for the decision between productive or latent infection following viral entry into neurons has yet to be fully defined. HSV immediate early protein ICP0 can ubiquitinate host proteins to target them for proteasomal degradation, providing a mechanism by which HSV could remove host proteins detrimental to HSV infection. Therefore, we sought to identify proteins targeted for ubiquitination by ICP0 during productive infection, specifically in primary adult sensory DRG neurons, using a comparative mass spectrometry approach. We identified both host and viral proteins that were ubiquitinated by ICP0, as well as proteins that were ubiquitinated by both ICP0 and host processes. These proteins likely play key roles in early events during HSV-1 neuronal infection.

Ubiquitination of proteins is complex and can have multiple downstream effects, depending on the type of ubiquitin moieties present on the target protein. Mono-ubiquitination typically has a low affinity for the proteasome but often alters the subcellular location or function of the target protein [66]. Poly-ubiquitination, specifically K11 and K48, often results in degradation of the target protein by proteasomes [67]. K48 and K63 can also lead to phagolysosome autophagic degradation [68]. These outcomes are further nuanced by functional overlap and mixed or branched polyubiquitin chains that could combine multiple ubiquitin moieties with elements of K11, K29, K48, and K63 chains. K29 chains, for example, are often found in combination with other ubiquitin moieties, and its role in protein functions remains unclear, in part due to a lack of sufficient antibodies to study its effects in isolation [69]. We did not attempt to identify specific ubiquitin chains on the proteins we identified in this study, as ICP0 has been reported to stimulate the formation of complex ubiquitin chains in vitro [28], and the direct biological implications of this in neurons is unclear.

Previous reports have supported the ubiquitination of ICP0 in cell-free assays, suggesting this post-translational modification would facilitate its proteasomal degradation [50]. In our studies, we identified ICP0 in our original mass spectrometry analysis using the anti-ubiquitin antibody FK2 to pull out ubiquitinated proteins, suggesting that it was indeed ubiquitinated during productive infection in sensory neurons. Although we did not observe patterns of ubiquitination for ICP0 by immunoblot, ICP0 was degraded by the proteasome, as shown by the use of a proteasome inhibitor. As a RING finger E3-ubiquitin ligase, ICP0, remaining true to classification, would be prone to auto-ubiquitination; however, auto-ubiquitination is tightly controlled and more prevalent in the absence of other target substrate proteins [70,71,72]. The lack of observable bands indicative of auto-ubiquitination could be the result of target/substrate abundance during the initial infection of HSV-1 [50,72]. In addition, ICP0 has been reported to rely on host protein USP7 to protect itself from autoubiquitination while remaining active to target additional substrates during productive infection [50]. The antibody binding epitope is also near the ubiquitination site. Thus, protein–protein interaction between ICP0 and other proteins or post-translational modifications could prevent the detection of ubiquitinated ICP0 by masking the antibody binding site. These observations do not eliminate the possibility of ubiquitin-independent mechanisms causing ICP0 degradation, as previously observed in human embryonic lung fibroblasts during early HSV-1 infection [73].

ICP0 has been shown to target key proteins such as nuclear antigen Sp100 [36], other nuclear domain 10 (ND10) components [74], p53 [30], and Schlafen 5 [24], but we did not identify these proteins in our LC-MS/MS analysis. These differences in ICP0 ubiquitination targets are most likely due to differences in how primary sensory neurons regulate HSV-1 infection in comparison to non-neuronal cells. Any antiviral response that would likely induce apoptosis in infected non-neuronal cells must restrict viral replication and promote survival of neurons, as neurons are rather important and not easily replaced. We did, however, identify immediate-early, early, and late HSV-1 genes in KOS-infected neurons, suggesting ICP0 mediates the ubiquitination and degradation of viral proteins. These proteins included all temporal classes of viral genes, which would be expected to be expressed in KOS-infected neurons but not necessarily ubiquitinated and targeted for degradation. ICP4, VP16, major DNA binding protein (ICP8), thymidine kinase, and even ICP0 are known to promote conditions for lytic infection, activate viral gene expression, and mediate viral replication [75,76,77,78,79,80]. Structural components of the virus, including capsid proteins and envelope glycoproteins, were also identified. Taken together, these results suggest that ICP0 may actively target viral lytic proteins for ubiquitination early during infection of sensory neurons to induce an abortive infection, promoting the establishment of latency. Although this is contradictory to the accepted role of ICP0 stimulating viral transcription, the identification of fifteen lytic viral proteins ubiquitinated by ICP0 suggests that ICP0 may have different functions in non-neuronal and neuronal cells. Alternatively, ICP0 may restrict viral replication in some sub-populations of sensory neurons.

HMG I/Y is a transcriptional regulator that binds the minor groove of DNA, making it more accessible to RNA polymerase and transcription factors [41,44]. As an architectural transcription factor, HMG I/Y has no standard transcriptional function but is highly dependent on cofactors for its target specificity [43], serving dual functions as an activator of viral and host antiviral genes [81]. Dependence of HMG I/Y on cofactors results in complex and diverse regulatory mechanisms at transcriptional [82] and translational levels by proteins, pseudogenes, and microRNAs, in addition to post-translational modifications [83]. HMG I/Y has been shown to interact with promoters of HSV-1 genes, such as ICP4 and the latency-associated transcript (LAT) [84]. HMG I/Y has also been reported to enhance gene transcription of other viruses, including human papillomavirus (HPV) [85] and hepatitis B virus (HBV) [86]. In contrast, HMG I/Y has been implicated in enhancing the expression of interferon genes that restrict viral transcription [87]. This creates a complex interaction with HMG I/Y-regulated host genes and viral genes. In our studies, HMG I/Y expression initially increased in HSV-1 KOS-infected neurons but not in *n*212-infected neurons. HMG I/Y is reportedly sustained during HPV infection to aid in the coordination of gene expression [85,88] and is directly upregulated by HBV X protein [86], so HMG I/Y may be necessary for some aspect of HSV-1 infection. HMG I/Y may have a transient enhancer and subsequent inhibitory role to HSV-1 in neurons depending on the viral proteins present, such as ICP4 [45]. However, ubiquitination by ICP0 and degradation of HMG I/Y by 10 h after infection could suggest a shift in viral mechanics, such that ICP0 attempts to abort a productive infection and facilitate establishment of latency.

In contrast to HMG I/Y, TDP43 expression was reduced in the presence of functional ICP0 during productive infection. Although TDP43 was ubiquitinated by ICP0, it was not degraded in the ubiquitin-proteasome, suggesting that TDP43 may have been functionally redirected or degraded through some other process. TDP43 is a nucleic acid binding protein that was first identified as an HIV viral repressor [46]. More recently, TDP43 has been implicated in gene regulation [89], RNA metabolism, tissue-specific gene expression [47,90], and modulation of stress granules in response to cellular stress [91]. In normal homeostatic tissue, TDP43 modulates the stability and decay of host mRNA [47,48], impacting up to 30% of the transcriptome [89]. Its broad range allows it to influence many biological activities. For example, TDP43 stabilizes the mRNA of Ras-GAP SH3-domain binding protein 1 (G3BP1) [92], a key stress granule protein that promotes efficient activation of cyclic GMP-AMP synthase (cGAS), an important innate sensor directed against DNA viruses to activate a type-1 interferon response [93,94]. G3BP1 mRNA is reduced when TDP43 protein levels are low, as we found during productive infection in neurons infected with KOS, suggesting this could be a potential mechanism utilized by HSV-1 to control the host antiviral response.

This study presents unique insight into the manipulation of the neuronal environment of primary adult sensory neurons through ICP0-mediated ubiquitination of host cell proteins during HSV-1 infection. For productive infection in epithelial cells, ICP0 engages in dynamic protein–protein interactions with multiple proteins to enhance viral replication. These interactions are often dominated by ICP0′s ubiquitination function to create a cellular environment conducive for the production of viral progeny. In neurons, ubiquitination of host proteins could facilitate either productive infection or the establishment of latency, from which it can later reactivate to cause recurrent disease and transmit to new hosts. Our results, showing that ICP0 ubiquitinates and facilitates degradation of key host proteins at specific times following infection, suggest an exquisitely complex manipulation of the neuronal environment. We did not detect proteins that were previously identified as ICP0 ubiquitination targets in non-neuronal cells, such as ND10 proteins or regulators of the interferon response, which would inhibit productive infection. We did, however, detect multiple HSV-1 proteins in our mass spectrometry screen, strongly suggesting that ICP0 actively targets its own productive cycle proteins for ubiquitination to potentially facilitate the establishment of latency in sensory neurons. Although further studies are needed to fully define the sequence of events orchestrated by ICP0 in neurons, the targets of ICP0 appear to be distinct from those in non-neuronal cells.

## 4. Conclusions and Future Studies

ICP0 and its targets have been studied extensively in non-neuronal cells, but its neuron-specific targets remain largely unknown. HSV-1 immediate early protein ICP0 catalyzes the transfer of ubiquitination to target proteins, allowing HSV-1 to control and modulate the neuronal environment during infection. This study identifies HMG I/Y, TDP43, and HSV-1 viral proteins as ICP0 ubiquitination targets during acute infection in primary mature sensory neurons. Other putative target proteins were also identified, but will require further study. The results of our comparative proteomics approach suggest potential host anti-viral and viral anti-host mechanisms, emphasizing the complexity of HSV neuronal infection. In addition, ICP0 ubiquitinates HSV lytic proteins, which may attenuate their functions. Future studies will further investigate the sequence of events that govern the decision between lytic and latent infection in sensory neurons. Understanding these events may lead to the development of targeted antivirals to inhibit HSV-1 lytic infection.

## 5. Materials and Methods

### 5.1. Cells and Viruses

Vero76 cells (C1008, ATCC, Manassas, VA, USA) were maintained in Dulbecco’s Modified Eagle Medium (DMEM; Thermo Fisher Scientific, Waltham, MA, USA), supplemented with 8% fetal bovine serum (FBS) and 1% penicillin-streptomycin (PS). HSV-1 KOS (wild-type) and *n*212 strains [95] (both originally from the laboratory of Priscilla Schaefer) were generously provided to the Bertke Lab by David Davido at the University of Kansas Department of Molecular Biosciences, Lawrence, Kansas, USA. HSV-1 *n*212 expresses a truncated ICP0 with a nonsense linker insertion at amino acid 212 on a KOS backbone [38]. KOS was propagated and titrated on Vero76 cells and *n*212 on L7 cells [96] that express complementing ICP0, also kindly provided by David Davido.

### 5.2. Primary Adult Neuronal Cultures

Dorsal root ganglia (DRG) were resected from 6-week-old Swiss Webster mice (Hilltop Laboratories, Scottdale, PA, USA) and enzymatically digested using papain and collagenase/dispase (Worthington Biochemical, Lakewood, NJ, USA), followed by mechanical separation into single-cell suspensions. Neurons were counted and plated on Matrigel-coated (Corning, Silicon Valley, CA, USA) cell culture plates at 3000–80,000 neurons per well, depending on the assay [9]. DRG neurons were maintained in Neurobasal A medium (Thermo Fisher Scientific, Waltham, MA, USA) with 1% penicillin-streptomycin (PS), 1× Glutamax, DNA synthesis inhibitor 5-fluoro-2′deoxyuridine (FUDR) to deplete non-neuronal cells by mitotic inhibition, and neurotrophic factors (nerve growth factor, glial cell-derived neurotrophic factor, neurturin; obtained from PeproTech, Cranbury, NJ, USA) [9]. DRGs were allowed to acclimate to the culture plate for 3–4 days before experimental procedures. All studies were conducted in accordance with the Institutional Animal Care and Use Committee at Virginia Tech (protocol approved 8 February 2019).

### 5.3. Infection

Maintenance media was removed and DRG neuronal cultures or Vero 76 cells were inoculated with 30 multiplicity of infection (MOI) of HSV-1 KOS or *n*212 viruses in Neurobasal A medium (Thermo Fisher Scientific) for 1 h. The viral inoculum was subsequently removed and replaced with NeuroComp media (Neurobasal A medium with 1% PS, Glutamax, and neurotrophic factors, but no FUDR) and maintained for the time periods indicated in the figures.

### 5.4. Antibodies

Primary antibodies included HSV-1 ICP0 (11060, Santa Cruz Biotechnology, Santa Cruz, CA, USA), TDP43 (GTX114210, GeneTex, Irvine, CA, USA and ab1044223, Abcam, Waltham, MA, USA), HMG I/Y (393213, Santa Cruz and ab168260, Abcam), and anti-ubiquitin FK2 (BML-PW8810-0500, Enzo Biochem, Farmingdale, NY, USA or Sigma Aldrich, St. Louis, MO, USA). Primary antibodies were visualized with secondary antibody goat anti-mouse or goat anti-rabbit IgG-HRP (31430 and 31460, Thermo Fisher Scientific).

### 5.5. LC-MS/MS

A total of 80,000 cultured DRG neurons per treatment (40,000 neurons/well and two wells were combined for each experiment) were infected with HSV-1 KOS or HSV-1 *n*212, or were uninfected, and treated with MG132 (Cbz-Leu-Leu-Leucinal) to inhibit the ubiquitin-proteasomal degradation complex and preserve ubiquitinated proteins post-inoculation. The infection progressed for 8 h prior to protein harvesting in 125 μL non-denaturing lysis buffer (20 mM Tris HCl pH8, 1% NP-40, 2 mM EDTA) with MG132, PR619 (2,6-Diaminopyridine-3,5-bis(thiocyanate)) broad-spectrum deubiquitinating enzyme inhibitor to prevent the removal of ubiquitin moieties after collection, and Halt Protease & Phosphatase Inhibitor Cocktail (Thermo Fisher Scientific). Samples were incubated for 12 h with FK2 anti-ubiquitin antibody covalently conjugated to Invitrogen Dynabeads (Thermo Fisher Scientific), according to the manufacturer’s protocol. Immunoprecipitated samples were rinsed 3 times with non-denaturing buffer and resuspended in non-denaturing lysis buffer with MG132, PR619, and Halt Protease & Phosphatase Inhibitor. Samples were shipped overnight to MSBioworks (Ann Arbor, MI, USA) for LC-MS/MS analysis. Each sample was eluted in 70 μL 1.5× NuPage LDS Sample Buffer (Thermo Fisher Scientific) and boiled at 100 °C for 15 min, followed by clarification via centrifugation. Half of each sample was processed by SDS-PAGE using a 10% Bis-Tris NuPage Mini-gel (Thermo Fisher Scientific) with an MES buffer system. A 2 cm gel space was excised into ten bands, washed with 25 mM ammonium bicarbonate and acetonitrile, reduced with 10 mM dithiothreitol at 60 °C, alkylated with 50 mM iodoacetamide at room temperature, digested with trypsin at 37 °C for 4 h, quenched with formic acid, and finally analyzed using a nano LC-MS/MS with Waters M-Class LC system interfaced to a Fusion Lumos mass spectrometer (Thermo Fisher Scientific). Each sample was analyzed for 5 h. The infection was repeated a second time, using neuronal cultures performed on a different day and following the identical processes and protocols to generate a replicate set of mass spectrometry data.

### 5.6. Data Processing

Raw data files from MSBioworks were downloaded by Virginia Tech Mass Spectrometry Incubator and reprocessed using Proteome Discoverer v. 2.5 (Thermo Fisher Scientific). Data files corresponding to all 10 bands of the same sample type were analyzed together. Searches using both Mascot (Matrix Science, Mount Prospect, IL, USA) and SequestHT (Thermo Fisher Scientific) were performed against the herpes simplex virus 1 reference proteome downloaded from UniProt, the mouse reference proteome downloaded from UniProt, and a database containing a list of common contaminant proteins provided with the Proteome Discoverer software, and the results merged using Proteome Discoverer. Search parameters included trypsin specificity with the possibility of two missed cleavages, precursor mass tolerance of ±10 ppm, fragment mass tolerance of ±0.1 Da, fixed modification of carbamidomethylation of Cys residues, and the following variable modifications: oxidation of Met, deamidation of Asn and Gln, formation of pyro-Glu when Gln was at the C-terminus of a peptide, acetylation of the protein N-terminus, and GlyGly characteristic of ubiquitin after trypsin cleavage at Lys and the protein N-terminus. The IMP-ptmRS node within Proteome Discoverer was utilized to validate the position and level of confidence for the GlyGly modification. The two repetitions were analyzed separately, and proteins identified in both replicates were included in the final comparative analyses.

### 5.7. Statistical Analysis

LC-MS/MS proteins were identified using a minimum of 2 unique peptide sequences and a 1% false discovery rate (FDR). Common contaminants and identified proteins with a MASCOT score < 50 (*p* < 0.00001 compared to average MASCOT score of each dataset) and an Exp-q value ≥ 0.05 were manually removed, and the resulting list was collated for comparison. The relative abundance for each protein was determined based on the peptide spectrum match (PSM) normalized to total spectra for each run provided by MSBioworks. Normalized PSM from each replicate were averaged within the respective treatment and compared to the other treatments by ANOVA and posthoc Dunnet’s test using KOS as the control group in JMP Pro 16 (SAS Institute, Cary, NC, USA). Proteins with a minimum of 1.25-fold increase in HSV-1 KOS samples, compared to both HSV-1 *n*212 and uninfected controls, or had a *p* < 0.05 by Dunnet’s, were selected for downstream biological justification. A literature search was performed on the selected proteins for previously reported functions to identify specific proteins with potential relevance to HSV-1 infection in neurons. Viral proteins that were identified by mass spectrometry were screened at a minimum MASCOT score of 30 (*p* < 0.01), since viral proteins were expected to be present at low concentrations compared to total protein. Densitometry statistics were performed on three biological replicates from different neuronal cultures and immunoblots. Protein of interest bands were quantified using Image Studio Lite version 5.2 (Li-Cor Biosciences, Lincoln, NE, USA) to calculate optical density of the protein band normalized to the optical density of the total protein in the relevant lane, visualized using 2,2,2-trichloroethanol (TCE), a stain-free, fluorescent method of total protein detection. Optical densities were quantified twice for each blot using different exposures, and averaged. Standard error of the means and ANOVAs were performed using JMP Pro 16.

### 5.8. Immunoblot

Neuronal cells were collected in 100 μL radioimmunoprecipitation assay (RIPA) buffer. Sample protein concentrations were quantified using Quick Start Bradford Dye Reagent (BioRad, Philadelphia, PA, USA) and Bovine Serum Albumin Standard Set (BioRad). A total of 15 μg of protein were loaded into a 10–12.5% sodium dodecyl sulfate-polyacrylamide (SDS-PAGE) gel with 1% 2,2,2-trichloroethanol (TCE) to measure total protein and validate equal loading by stain-free fluorescence. Proteins were subsequently transferred to polyvinylidene difluoride (PVDF) transfer membrane (Millipore, Burlington, MA, USA) and blocked in 5% milk buffer. Membranes were incubated with 5–8 μg of primary antibody for 4 h (HMG I/Y, TDP43) or overnight (ICP0) and 2.4 μg of HRP-conjugated secondary antibody for 2 h. Blots were imaged by chemiluminescence using SuperSignal West Femto Maximum Sensitivity Substrate (Thermo Fisher Scientific). Protein bands were analyzed by densitometry and normalized to total protein in each lane, based on TCE densitometry analysis.

### 5.9. Immunoprecipitation

Neuronal cells were collected in 125 μL non-denaturing lysis buffer (20 mM Tris HCl pH8, 1% NP-40, 2 mM EDTA) with MG132 (Cbz-Leu-Leu-Leucinal) to inhibit the proteasome, PR619 (2,6-Diaminopyridine-3,5-bis(thiocyanate)) broad-spectrum deubiquitinating enzyme inhibitor to prevent the removal of ubiquitin moieties after collection, and Halt Protease & Phosphatase Inhibitor (Thermo Fisher Scientific). Sample protein concentrations were quantified using Quick Start Bradford Dye Reagent (BioRad) and Bovine Serum Albumin Standard Set (BioRad). Immunoprecipitation was performed by covalently conjugating 5 μg of antibody to 1 mg Invitrogen Dynabeads (Thermo Fisher Scientific) using Dynabeads Antibody Coupling Kit (Thermo Fisher Scientific) according to the manufacturer’s protocol. A total of 15 μg DRG total protein was incubated with antibody-coupled Dynabeads for 12 h at 4 °C. Immunoprecipitated samples were rinsed with non-denaturing buffer and resuspended in a mixture of 25 μL non-denaturing buffer, 10 μL 4× Laemmli buffer (with 10% BME) (total volume of 35 μL) heated to 95 °C and loaded into a 10–12.5% SDS-PAGE gel for Western blot. Total protein as input protein control (InP), supernatant from antibody-bead conjugate supernatant (Sup), and antibody-bead eluate (IP) were loaded to ensure no antibody leeching and successful immunoprecipitation.

## Figures and Tables

**Table 1:** Virus and host proteins identified by LC-MS/MS as ubiquitinated.

	Accession	Protein	Gene	UI	KOS	*n*212	KOS/UI	KOS/*n*212	KOS:UI (*p* Value)	KOS:*n*212 (*p* Value)
**Viral Proteins**	P03176	Thymidine kinase	TK	0.0	12.5	3.0	NC	4.17	0.0016	0.0136
	P08543	Ribonucleoside-diphosphate reductase large subunit	RIR1	0.0	112.0	3.5	NC	32.00	0.0249	0.0266
	P10221	Inner tegument protein	UL37	0.0	58.5	0.0	NC	NC	0.0018	0.0018
	P04296	Major DNA-binding protein	DBP	0.0	90.0	0.0	NC	NC	0.0202	0.0202
	P04294	Alkaline nuclease	UL12	0.0	41.0	0.0	NC	NC	0.0279	0.0279
	P10211	Envelope glycoprotein B	gB	0.0	22.0	0.0	NC	NC	0.0670	0.0670
	P04485	Transcriptional regulator ICP22	ICP22	0.0	21.0	0.0	NC	NC	0.0955	0.0955
	P06492	Tegument protein VP16	UL48	0.0	21.0	0.0	NC	NC	0.1196	0.1196
	P08392	Major viral transcription factor ICP4	ICP4	0.0	28.0	0.0	NC	NC	0.2222	0.2222
	P06491	Major capsid protein	MCP	0.0	46.0	0.0	NC	NC	0.2421	0.2421
	P08393	E3 ubiquitin-protein ligase ICP0	ICP0	0.0	36.0	0.0	NC	NC	0.2865	0.2865
	P04488	Envelope glycoprotein E	gE	0.0	6.5	0.0	NC	NC	0.4226	0.4226
**Host Proteins**	Q60900	ELAV-like protein 3	Elavl3	0.0	21.0	0.0	NC	NC	0.0023	0.0023
	P61027	Ras-related protein Rab-10	Rab10	0.0	20.0	0.0	NC	NC	0.0025	0.0025
	A0A1B0GS70	Proteasome endopeptidase complex	Psma1	0.0	15.5	0.0	NC	NC	0.0250	0.0250
	P54775	26S proteasome regulatory subunit 6B	Psmc4	0.0	21.5	0.0	NC	NC	0.0255	0.0255
	Q9JKC6	Cell cycle exit and neuronal differentiation protein 1	Cend1	0.0	12.0	0.0	NC	NC	0.0572	0.0572
	P49312	Heterogeneous nuclear ribonucleoprotein A1	Hnrnpa1	0.0	19.0	0.0	NC	NC	0.0628	0.0628
	P17095	High mobility group protein HMG-I/HMG-Y	Hmga1	0.0	12.5	0.0	NC	NC	0.4226	0.4226
	P43274	Histone H1.4	H1-4	0.0	72.5	21.5	NC	3.37	0.2593	0.3874
	P15864	Histone H1.2	H1-2	0.0	74.5	23.0	NC	3.24	0.3050	0.4444
	Q91ZZ3	Beta-synuclein	Sncb	0.0	40.0	18.0	NC	2.22	0.0056	0.0258
	Q8R0B4	TAR DNA-binding protein 43	Tardp	0.0	29.0	15.5	NC	1.87	0.0105	0.0995
	Q3THW5	Histone H2A.V	H2az2	0.0	41.0	23.5	NC	1.74	0.0450	0.1917

Note: Complete list of identified proteins can be found in Supplementary Table S1.

## Data Availability

Mass spectrometry proteomics data were deposited to the ProtomeXchange Consortium via the PRIDE [97] partner repository, with the data set identifier PXD037767 and 10.6019/PXD037767.

## Supplementary Materials

The following supporting information can be downloaded at: https://www.mdpi.com/article/10.3390/ijms24032931/s1.

## Institutional Animal Care and Use Statement

The study was conducted in accordance with the Office of Laboratory Animal Welfare, and the animal study protocol was approved by the Institutional Review Board of Virginia Polytechnic Institute and State University (protocol 18-274 approved 8 February 2019 and 21-244 approved 11 January 2022).

## Abbreviations

**Abbreviation**	**Definition**
HSV-1	Herpes simplex virus 1
KOS	HSV-1 wildtype strain KOS
*n*212	HSV-1 strain *n*212, defective ICP0 protein
UI	Uninfected
hpi	Hours post inoculation
IE	Immediate early
ICP0	Infected cell protein 0
ICP22	infected cell protein 22
ICP27	Infected cell protein 27
ICP47	Infected cell protein 47
VP16	HSV tegument viral protein 16, trans-inducing protein
TK	HSV thymidine kinase
DRG	Dorsal root ganglion
HMG I/Y	High mobility group protein I/Y
TDP43	TAR-DNA binding protein 43
USP7	Ubiquitin-specific peptidase 7
UbcH5a	Ubiquitin-conjugating enzyme H5a
UbcH6a	Ubiquitin-conjugating enzyme H6a
PML	Promyelocytic leukemia protein
LC-MS/MS	Nano liquid chromatography tandem mass spectrometry
PSM	Peptide spectrum matches
TCE	2,2,2-Trichloroethanol
IP	Immunoprecipitation
InP	Input
