# Supplementary material for: Herpes Simplex Virus 1 (HSV-1) Infected Cell Protein 0 (ICP0) Targets of Ubiquitination during Productive Infection of Primary Adult Sensory Neurons
Source: Int J Mol Sci. 2023 Feb 2;24(3):2931. doi: 10.3390/ijms24032931 (PMC9917815; doi:10.3390/ijms24032931)
Supplement: Supplementary file 1 [file ijms-24-02931-s001.zip › ijms-2120913-Table S1.pdf]

Accession	Protein	Gene	UI	KOS	n212	KOS/ UI	KOS/ n212	KOS:UI (*p* value)	KOS:n212 (*p* value)
**Viral Proteins**									
P03176	Thymidine kinase	TK	0.0	12.5	3.0	NC	4.17	0.0016	0.0136
P08543	Ribonucleoside-diphosphate reductase large subunit	RIR1	0.0	112.0	3.5	NC	32.00	0.0249	0.0266
P10221	Inner tegument protein	UL37	0.0	58.5	0.0	NC	NC	0.0018	0.0018
P04296	Major DNA-binding protein	DBP	0.0	90.0	0.0	NC	NC	0.0202	0.0202
P04294	Alkaline nuclease	UL12	0.0	41.0	0.0	NC	NC	0.0279	0.0279
P10202	Triplex capsid protein 2	TRX2	0.0	10.5	0.0	NC	NC	0.0523	0.0523
P10205	Tegument protein UL21	UL21	0.0	5.5	0.0	NC	NC	0.0670	0.0670
P10211	Envelope glycoprotein B	gB	0.0	22.0	0.0	NC	NC	0.0670	0.0670
P04485	Transcriptional regulator ICP22	ICP22	0.0	21.0	0.0	NC	NC	0.0955	0.0955
P06492	Tegument protein VP16	UL48	0.0	21.0	0.0	NC	NC	0.1196	0.1196
P10209	Capsid vertex component 2	CVC2	0.0	10.0	0.0	NC	NC	0.1296	0.1296
P10238	mRNA export factor	UL54	0.0	17.0	0.0	NC	NC	0.1358	0.1358
P08392	Major viral transcription factor ICP4	ICP4	0.0	28.0	0.0	NC	NC	0.2222	0.2222
P06491	Major capsid protein	MCP	0.0	46.0	0.0	NC	NC	0.2421	0.2421
P32888	Triplex capsid protein 1	TRX1	0.0	11.0	0.0	NC	NC	0.2567	0.2567
P08393	E3 ubiquitin-protein ligase ICP0	ICP0	0.0	36.0	0.0	NC	NC	0.2865	0.2865
P04488	Envelope glycoprotein E	gE	0.0	6.5	0.0	NC	NC	0.4226	0.4226
**Host Proteins Only in KOS-Infected Neurons**									
Q91V41	Ras-related protein Rab-14	RAB14	0.0	17.5	0.0	NC	NC	0.0008	0.0008
P23927	Alpha-crystallin B chain	Cryab	0.0	16.5	0.0	NC	NC	0.0009	0.0009
P20029	Endoplasmic reticulum chaperone BiP	Hspa5	0.0	32.0	0.0	NC	NC	0.0010	0.0010
Q9DCX2	ATP synthase subunit d, mitochondrial	Atp5pd	0.0	15.5	0.0	NC	NC	0.0010	0.0010
Q60900	ELAV-like protein 3	Elavl3	0.0	21.0	0.0	NC	NC	0.0023	0.0023
P61027	Ras-related protein Rab-10	Rab10	0.0	20.0	0.0	NC	NC	0.0025	0.0025
F6RBR6	NADH dehydrogenase [ubiquinone] 1 alpha subcomplex subunit 12	Ndufa12	0.0	8.5	0.0	NC	NC	0.0034	0.0034
A2AKV1	ATP synthase, H+-transporting, mitochondrial F1 complex, gamma polypeptide 1	Atp5c1	0.0	53.5	0.0	NC	NC	0.0070	0.0070
Q923T9	Calcium/calmodulin-dependent protein kinase type II subunit gamma	Camk2g	0.0	9.0	0.0	NC	NC	0.0121	0.0121
Q7TMM9	Tubulin beta-2A chain	Tubb2a	0.0	62.0	0.0	NC	NC	0.0125	0.0125
Q8C181	Muscleblind-like protein 2	Mbnl2	0.0	10.5	0.0	NC	NC	0.0198	0.0198
P56382	ATP synthase subunit epsilon, mitochondrial	Atp5f1e	0.0	13.0	0.0	NC	NC	0.0229	0.0229
P53994	Ras-related protein Rab-2A	Rab2a	0.0	9.5	0.0	NC	NC	0.0240	0.0240
A0A1B0GS70	Proteasome endopeptidase complex	Psma1	0.0	15.5	0.0	NC	NC	0.0250	0.0250
P54775	26S proteasome regulatory subunit 6B	Psmc4	0.0	21.5	0.0	NC	NC	0.0255	0.0255
P56399	Ubiquitin carboxyl-terminal hydrolase 5	Usp5	0.0	13.5	0.0	NC	NC	0.0326	0.0326

P15331-3	Isoform 5b of Peripherin	Prph	0.0	247.5	0.0	NC	NC	0.0392	0.0392
P35486	Pyruvate dehydrogenase E1 component subunit alpha, somatic form, mitochondrial	Pdha1	0.0	14.5	0.0	NC	NC	0.0536	0.0536
Q9JKC6	Cell cycle exit and neuronal differentiation protein 1	Cend1	0.0	12.0	0.0	NC	NC	0.0572	0.0572
P49312	Heterogeneous nuclear ribonucleoprotein A1	Hnrnpa1	0.0	19.0	0.0	NC	NC	0.0628	0.0628
Q8BG05-2	Isoform 2 of Heterogeneous nuclear ribonucleoprotein A3	Hnrnpa3	0.0	45.0	0.0	NC	NC	0.0643	0.0643
Q7TMY8	E3 ubiquitin-protein ligase HUWE1	Huwe1	0.0	23.5	0.0	NC	NC	0.0687	0.0687
P35700	Peroxiredoxin-1	Prdx1	0.0	17.0	0.0	NC	NC	0.1053	0.1053
Q9D6R2	Isocitrate dehydrogenase [NAD] subunit alpha, mitochondrial	Idh3a	0.0	23.5	0.0	NC	NC	0.1097	0.1097
Q61171	Peroxiredoxin-2	Prdx2	0.0	17.5	0.0	NC	NC	0.1147	0.1147
Q61990	Poly(rC)-binding protein 2	Pcbp2	0.0	19.0	0.0	NC	NC	0.1692	0.1692
Q9QZQ8	Core histone macro-H2A.1	Macroh2a1	0.0	26.0	0.0	NC	NC	0.2044	0.2044
Q9D0E1	Heterogeneous nuclear ribonucleoprotein M	Hnrnpm	0.0	67.0	0.0	NC	NC	0.2119	0.2119
P62259	14-3-3 protein epsilon	Ywhae	0.0	12.5	0.0	NC	NC	0.2375	0.2375
P17095	High mobility group protein HMG-I/HMG-Y	Hmga1	0.0	12.5	0.0	NC	NC	0.4226	0.4226
**Host Proteins Only in Infected Neurons (KOS and n212)**									
P43274	Histone H1.4	H1-4	0.0	72.5	21.5	NC	3.37	0.2593	0.3874
P15864	Histone H1.2	H1-2	0.0	74.5	23.0	NC	3.24	0.3050	0.4444
Q9DCN2	NADH-cytochrome b5 reductase 3	Cyb5r3	0.0	20.0	7.0	NC	2.86	0.0218	0.0544
A6ZI44	Fructose-bisphosphate aldolase	Aldoa	0.0	32.0	13.5	NC	2.37	0.0572	0.1683
Q91ZZ3	Beta-synuclein	Sncb	0.0	40.0	18.0	NC	2.22	0.0056	0.0258
P12787	Cytochrome c oxidase subunit 5A, mitochondrial	Cox5a	0.0	8.5	4.0	NC	2.13	0.0767	0.2951
P08113	Endoplasmin	Hsp90b1	0.0	23.0	11.5	NC	2.00	0.0166	0.1301
Q64524	Histone H2B type 2-E	H2bc21	0.0	92.5	49.0	NC	1.89	0.1155	0.3370
Q8R0B4	TAR DNA-binding protein 43	Tardp	0.0	29.0	15.5	NC	1.87	0.0105	0.0995
P14602	Heat shock protein beta-1	Hspb1	0.0	52.0	28.5	NC	1.82	0.0741	0.2962
P48036	Annexin A5	Anxa5	0.0	18.0	10.0	NC	1.80	0.0031	0.0572
Q3THW5	Histone H2A.V	H2az2	0.0	41.0	23.5	NC	1.74	0.0450	0.1917
G5E902	Phosphate carrier protein, mitochondrial	lc25a3	0.0	30.5	17.5	NC	1.74	0.0425	0.1907
P48962	ADP/ATP translocase 1	Slc25a4	0.0	69.5	45.0	NC	1.54	0.0463	0.3264
Q64523	Histone H2A type 2-C	H2ac20	0.0	52.5	34.5	NC	1.52	0.0312	0.2084
P14873	Microtubule-associated protein 1B	Map1b	0.0	34.5	23.0	NC	1.50	0.2649	0.6703
P30275	Creatine kinase U-type, mitochondrial	Ckmt1	0.0	10.0	7.0	NC	1.43	0.1296	0.5425
Q61425	Hydroxyacyl-coenzyme A dehydrogenase, mitochondrial	Hadh	0.0	29.0	21.0	NC	1.38	0.0047	0.4770
Q9DC70	NADH dehydrogenase [ubiquinone] iron-sulfur protein 7, mitochondrial	Ndufs7	0.0	5.5	4.0	NC	1.38	0.0082	0.0955
O08547	Vesicle-trafficking protein SEC22b	Seec22b	0.0	11.0	8.0	NC	1.38	0.0670	0.6094
Q9DB20	ATP synthase subunit O, mitochondrial	Atp5po	0.0	50.0	37.0	NC	1.35	0.0702	0.5288
P51881	ADP/ATP translocase 2	Slc25a5	0.0	53.0	39.5	NC	1.34	0.0276	0.3683

P68040	Receptor of activated protein C kinase 1	Rack1	0.0	18.0	14.0	NC	1.29	0.0460	0.5959
Q9CQQ7	ATP synthase F(0) complex subunit B1, mitochondrial	Atp5bp	0.0	36.0	28.5	NC	1.26	0.0121	0.6254
Q9D154	Leukocyte elastase inhibitor A	Serpinb1a	0.0	8.5	7.0	NC	1.21	0.0034	0.6707
P00405	Cytochrome c oxidase subunit 2	Mtco2	0.0	14.5	12.0	NC	1.21	0.0012	0.1548
Q99LC5	Electron transfer flavoprotein subunit alpha, mitochondrial	Etfa	0.0	9.5	8.0	NC	1.19	0.0240	0.6094
O55234	Proteasome subunit beta type-5	Psmb5	0.0	27.0	23.0	NC	1.17	0.0014	0.4343
O88569	Heterogeneous nuclear ribonucleoproteins A2/B1	Hnrnpa2b1	0.0	39.0	33.5	NC	1.16	0.0396	0.7012
P67778	Prohibitin	Phb	0.0	24.0	21.0	NC	1.14	0.0069	0.7758
E9Q3W4	Plectin	Plec	0.0	188.5	165.0	NC	1.14	0.0051	0.7638
Q9D1D4	Transmembrane emp24 domain-containing protein 10	Tmed10	0.0	20.5	18.5	NC	1.11	0.0006	0.1056
Q9Z2U1	Proteasome subunit alpha type-5	Psma5	0.0	12.5	11.5	NC	1.09	0.0141	0.8769
P49722	Proteasome subunit alpha type-2	Psma2	0.0	12.5	12.0	NC	1.04	0.0377	0.8698
G3UX26	Outer mitochondrial membrane protein porin 2	Vdac2	0.0	41.5	40.5	NC	1.02	0.0070	0.8769
O35129	Prohibitin-2	Phb2	0.0	36.0	35.5	NC	1.01	0.0121	0.9414
P55821	Stathmin-2	Stmn2	0.0	14.0	14.0	NC	1.00	0.0430	1.0000
Q9QUM9	Proteasome subunit alpha type-6	Psma6	0.0	26.0	28.0	NC	0.93	0.0015	0.8272
P99026	Proteasome subunit beta type-4	Psmb4	0.0	12.5	14.5	NC	0.86	0.0377	0.7351
Q60692	Proteasome subunit beta type-6	Psmb6	0.0	12.5	15.0	NC	0.83	0.0016	0.4975
G3UYU4	Flotillin	Flot1	0.0	9.5	12.0	NC	0.79	0.0628	0.4226
J3QMG3	Voltage-dependent anion-selective channel protein 3	Vdac3	0.0	21.0	27.0	NC	0.78	0.0955	0.4965
Q9R1P0	Proteasome subunit alpha type-4	Psma4	0.0	15.5	20.0	NC	0.78	0.0092	0.1299
O70435	Proteasome subunit alpha type-3	Psma3	0.0	24.0	32.0	NC	0.75	0.0017	0.6262
**Host Proteins Only in Uninfected Neurons**									
Q07076	Annexin A7	Anxa7	14.5	0.0	0.0	0.00	NC	0.0012	1.0000
Q91W39	Nuclear receptor coactivator 5	Ncoa5	12.0	0.0	0.0	0.00	NC	0.0267	1.0000
Q9D8W5	26S proteasome non-ATPase regulatory subunit 12	Psmd12	7.5	0.0	0.0	0.00	NC	0.0377	1.0000
P63268	Actin, gamma-enteric smooth muscle	Actg2	114.0	0.0	0.0	0.00	NC	0.0912	1.0000
P09405	Nucleolin	Ncl	39.5	0.0	0.0	0.00	NC	0.1125	1.0000
E9PV04	RNA helicase	Eif4a3l1	13.0	0.0	0.0	0.00	NC	0.1215	1.0000
A0A0R4J0B4	CMP-N-acetylneuraminic acid synthase	Cmas	14.5	0.0	0.0	0.00	NC	0.1554	1.0000
P57724	Poly(rC)-binding protein 4	Pcbp4	13.5	0.0	0.0	0.00	NC	0.1734	1.0000
P42669	Transcriptional activator protein Pur-alpha	Pura	14.5	0.0	0.0	0.00	NC	0.1929	1.0000
Q9Z2X1	Heterogeneous nuclear ribonucleoprotein F	Hnrnpf	23.5	0.0	0.0	0.00	NC	0.2009	1.0000
Q8VIJ6	Splicing factor, proline- and glutamine-rich	Sfpq	20.0	0.0	0.0	0.00	NC	0.2107	1.0000
Q8VEK3	Heterogeneous nuclear ribonucleoprotein U	Hnrnpu	47.5	0.0	0.0	0.00	NC	0.2487	1.0000
D3Z6E4	2-phospho-D-glycerate hydro-lyase	Eno2	18.5	0.0	0.0	0.00	NC	0.2770	1.0000
Q8BMK4	Cytoskeleton-associated protein 4	Ckap4	33.0	0.0	0.0	0.00	NC	0.2878	1.0000

Q70IV5	Synemin	Synm	56.5	0.0	0.0	0.00	NC	0.3065	1.0000
Q8BG05	Heterogeneous nuclear ribonucleoprotein A3	Hnrnpa3	41.5	0.0	0.0	0.00	NC	0.3067	1.0000
Q8C2Q3	RNA-binding protein 14	Rbm14	33.0	0.0	0.0	0.00	NC	0.3177	1.0000
A0A1L1STE6	Isocitrate dehydrogenase [NAD] subunit, mitochondrial	Idh3a	17.0	0.0	0.0	0.00	NC	0.3211	1.0000
Q3UVK0	Endoplasmic reticulum metallopeptidase 1	Ermp1	21.5	0.0	0.0	0.00	NC	0.3442	1.0000
O88398	Advillin	Avil	24.0	0.0	0.0	0.00	NC	0.3892	1.0000
**Host Proteins in All 3 Conditions**									
Q9Z0F7	Gamma-synuclein	Sncg	5.5	134.0	94.5	24.36	1.42	0.0057	0.1891
P10922	Histone H1.0	H1-0	16.0	59.5	20.0	3.72	2.98	0.4550	0.4769
Q8BWT1	3-ketoacyl-CoA thiolase, mitochondrial	Acaa2	21.5	31.5	25.5	1.47	1.24	0.5837	0.7430
P63038	60 kDa heat shock protein, mitochondrial	Hspd1	55.0	74.0	49.0	1.35	1.51	0.5369	0.5876
P17182	Alpha-enolase	Eno1	34.5	43.0	34.0	1.25	1.26	0.7146	0.6494
E9PUD2	Dynamin-1-like protein	Dnm1l	32.5	40.5	20.5	1.25	1.98	0.6790	0.1029
Q61937	Nucleophosmin	Npm1	41.5	51.5	20.5	1.24	2.51	0.8333	0.3768
P80314	T-complex protein 1 subunit beta	Cct2	50.0	58.5	44.0	1.17	1.33	0.2935	0.4837
P80315	T-complex protein 1 subunit delta	Cct4	37.0	42.5	26.5	1.15	1.60	0.8377	0.1179
O35857	Mitochondrial import inner membrane translocase subunit TIM44	Timm44	28.5	32.0	18.0	1.12	1.78	0.8868	0.6021
B1AXZ5	ELAV-like protein 2	Elavl2	33.0	35.5	24.5	1.08	1.45	0.8961	0.2929
D3Z4W5	RIKEN cDNA 1700074P13 gene (Fragment)	1700074P13Rik	36.5	39.0	66.5	1.07	0.59	0.8754	0.3186
P62192	26S proteasome regulatory subunit 4	Psmc1	45.0	44.0	31.5	0.98	1.40	0.9414	0.4239
P60843	Eukaryotic initiation factor 4A-I	Eif4a1	34.5	33.0	18.5	0.96	1.78	0.9520	0.3507
P62806	Histone H4	H4c1	197.0	184.0	138.5	0.93	1.33	0.9138	0.3135
Q61701	ELAV-like protein 4	Elavl4	35.5	33.0	23.0	0.93	1.43	0.8966	0.3406
Q99K48	Non-POU domain-containing octamer-binding protein	Nono	26.0	23.0	16.5	0.88	1.39	0.6335	0.3177
Q9ERD7	Tubulin beta-3 chain	Tubb3	94.0	75.0	63.5	0.80	1.18	0.8008	0.5161
P63017	Heat shock cognate 71 kDa protein	Hspa8	112.5	89.0	72.0	0.79	1.24	0.7046	0.5298
P99024	Tubulin beta-5 chain	Tubb5	107.5	83.0	83.5	0.77	0.99	0.7595	0.9749
Q60668	Heterogeneous nuclear ribonucleoprotein D0	Hnrnpd	15.0	11.5	7.0	0.77	1.64	0.7857	0.2366
P10126	Elongation factor 1-alpha 1	Eef1a1	81.5	62.0	76.5	0.76	0.81	0.7467	0.4830
P15331	Peripherin	Prph	341.5	252.5	202.5	0.74	1.25	0.7159	0.4703
E9Q5F6	Polyubiquitin-C (Fragment)	Ubc	598.5	440.0	455.5	0.74	0.97	0.5443	0.6564
P20152	Vimentin	Vim	353.0	256.5	207.0	0.73	1.24	0.7201	0.5934
Q8BVQ9	26S proteasome AAA-ATPase subunit RPT1	Psmc2	45.5	33.0	25.5	0.73	1.29	0.6440	0.6675
Q01853	Transitional endoplasmic reticulum ATPase	Vcp	65.5	47.0	48.0	0.72	0.98	0.7639	0.9645
O54734	Dolichyl-diphosphooligosaccharide--protein glycosyltransferase 48 kDa subunit	Ddost	28.0	20.0	15.0	0.71	1.33	0.6843	0.6324
Q8VDM4	26S proteasome non-ATPase regulatory subunit 2	Psmd2	71.0	50.0	38.5	0.70	1.30	0.4896	0.5981
P24668	Cation-dependent mannose-6-phosphate receptor	M6pr	25.5	17.5	15.0	0.69	1.17	0.5122	0.5984

A0A1B0GX81	BCL2-associated athanogene 6 (Fragment)	Bag6	27.0	18.5	18.5	0.69	1.00	0.4060	1.0000
Q03265	ATP synthase subunit alpha, mitochondrial	Atp5f1a	87.5	58.5	53.5	0.67	1.09	0.6937	0.5546
P63037	DnaJ homolog subfamily A member 1	Dnaja1	19.5	12.5	10.5	0.64	1.19	0.6888	0.8424
P56480	ATP synthase subunit beta, mitochondrial	Atp5f1b	65.0	41.5	35.0	0.64	1.19	0.6057	0.5457
Q9DB77	Cytochrome b-c1 complex subunit 2, mitochondrial	Uqcrc2	72.0	45.5	30.5	0.63	1.49	0.5530	0.3081
P62334	26S proteasome regulatory subunit 10B	Psmc6	35.0	22.0	16.0	0.63	1.38	0.6121	0.4929
Q8BG32	26S proteasome non-ATPase regulatory subunit 11	Psmd11	22.0	13.5	12.0	0.61	1.13	0.3712	0.8459
P08551	Neurofilament light polypeptide	Nefl	197.0	114.0	101.5	0.58	1.12	0.6094	0.7739
Q9CZ13	Cytochrome b-c1 complex subunit 1, mitochondrial	Ubcrc1	47.0	26.5	12.5	0.56	2.12	0.4550	0.2186
E9QNN1	RNA helicase	Dhx9	108.0	60.5	50.0	0.56	1.21	0.5915	0.8433
P11499	Heat shock protein HSP 90-beta	Hsp90ab1	68.0	37.0	47.5	0.54	0.78	0.6187	0.2233
P19246	Neurofilament heavy polypeptide	Nefh	118.0	64.0	58.5	0.54	1.09	0.5867	0.8592
Q99L43	Phosphatidate cytidylyltransferase 2	Cds2	19.0	10.0	9.5	0.53	1.05	0.5327	0.6985
Q60932-2	Isoform Mt-VDAC1 of Voltage-dependent anion-selective channel protein 1	Vdac1	84.0	42.0	48.5	0.50	0.87	0.6401	0.3588
Q62167	ATP-dependent RNA helicase DDX3X	Ddx3x	79.5	39.0	30.0	0.49	1.30	0.5618	0.6738
P63094	Guanine nucleotide-binding protein G(s) subunit alpha isoforms short	Gnas	35.5	16.0	17.5	0.45	0.91	0.5246	0.6335
P08553	Neurofilament medium polypeptide	Nefm	228.5	100.5	88.5	0.44	1.14	0.5872	0.8340
Q91YQ5	Dolichyl-diphosphooligosaccharide--protein glycosyltransferase subunit 1	Rpn1	65.5	28.5	18.5	0.44	1.54	0.5017	0.0422
Q9D394	Protein RUFY3	Rufy3	55.5	23.0	22.0	0.41	1.05	0.3590	0.9126
Q9D8N0	Elongation factor 1-gamma	Eef1g	63.0	26.0	18.0	0.41	1.44	0.5157	0.3037
P58252	Elongation factor 2	Eef2	53.0	15.5	9.0	0.29	1.72	0.4403	0.3524
P62814	V-type proton ATPase subunit B, brain isoform	Atp6v1b2	18.0	33.5	0.0	1.86	NC	0.4042	0.1312
P70333	Heterogeneous nuclear ribonucleoprotein H2	Hnrnph2	27.0	30.0	0.0	1.11	NC	0.8612	0.0044
Q8C2Q7	Heterogeneous nuclear ribonucleoprotein H	Hnrnph1	35.0	35.5	0.0	1.01	NC	0.9849	0.0157
P35564	Calnexin	Canx	25.0	21.0	0.0	0.84	NC	0.8272	0.0089
Q501J6	Probable ATP-dependent RNA helicase DDX17	Ddx17	83.5	64.0	0.0	0.77	NC	0.7494	0.1006
Q8BFU2	Histone H2A type 3	H2aw	70.5	50.5	0.0	0.72	NC	0.7795	0.0481
O08759	Ubiquitin-protein ligase E3A	Ube3a	22.5	15.0	0.0	0.67	NC	0.4192	0.0955
D3YUM1	NADH dehydrogenase [ubiquinone] flavoprotein 1, mitochondrial	Ndufv1	20.0	12.5	0.0	0.63	NC	0.6888	0.0377
O88685	26S proteasome regulatory subunit 6A	Psmc3	28.5	16.5	0.0	0.58	NC	0.3344	0.0009
P57722	Poly(rC)-binding protein 3	Pcbp3	35.0	19.5	0.0	0.56	NC	0.6140	0.2044
P46660	Alpha-internexin	Ina	50.0	27.0	0.0	0.54	NC	0.6137	0.0777
E9Q1W0	Calcium/calmodulin-dependent protein kinase	Camk2d	23.5	12.0	0.0	0.51	NC	0.5586	0.0069
H7BWX9	Small ubiquitin-related modifier 2	Sumo2	16.0	8.0	0.0	0.50	NC	0.3379	0.1835
Q9DBG6	Dolichyl-diphosphooligosaccharide--protein glycosyltransferase subunit 2	Rpn2	29.5	14.0	0.0	0.47	NC	0.5302	0.0198
Q91VD9	NADH-ubiquinone oxidoreductase 75 kDa subunit, mitochondrial	Ndufs1	49.5	23.0	0.0	0.46	NC	0.5886	0.0019
P60335	Poly(rC)-binding protein 1	Pcbp1	33.0	15.0	0.0	0.45	NC	0.5598	0.1654

Q8CAQ8	MICOS complex subunit Mic60	Immt	177.5	45.5	0.0	0.26	NC	0.0048	0.0332
P62196	26S proteasome regulatory subunit 8	Psmc5	46.0	0.0	32.0	0.00	0.00	0.0289	0.0447
P48678	Prelamin-A/C	Lmna	264.5	0.0	142.0	0.00	0.00	0.1290	0.1341
E9Q557	Desmoplakin	Dsp	45.5	0.0	89.0	0.00	0.00	0.1925	0.0011
Q8BFZ9	Erlin-2	Erlin2	25.0	0.0	16.0	0.00	0.00	0.2161	0.1835
Q02257	Junction plakoglobin	Jup	49.5	0.0	95.5	0.00	0.00	0.2354	0.0286
Q9D0I9	Arginine--tRNA ligase, cytoplasmic	Rars1	35.0	0.0	5.0	0.00	0.00	0.2675	0.0377
Q64475	Histone H2B type 1-B	H2bc3	114.0	0.0	72.5	0.00	0.00	0.2724	0.0004
